# Supplementary material for: Association of Tdap vaccine guidelines with vaccine uptake during pregnancy
Source: PLoS One. 2021 Jul 19;16(7):e0254863. doi: 10.1371/journal.pone.0254863 (PMC8289009; doi:10.1371/journal.pone.0254863)
Supplement: Attachment — Submitted filename: Responses to Reviewers.docx [file pone.0254863.s001.docx]

**EDITOR**

***Editor, Point 1:***

Please review your reference list to ensure that it is complete and correct. If you have cited papers that have been retracted, please include the rationale for doing so in the manuscript text, or remove these references and replace them with relevant current references. Any changes to the reference list should be mentioned in the rebuttal letter that accompanies your revised manuscript. If you need to cite a retracted article, indicate the article’s retracted status in the References list and also include a citation and full reference for the retraction notice.

***Response*:** We have reviewed our reference list and determined that none of the cited papers have been retracted.

***Editor, Point 2:***

Please ensure that your manuscript meets PLOS ONE's style requirements, including those for file naming.

***Response*:** We have updated the manuscript and file naming in accordance with the PLOS ONE’s style requirements.

***Editor, Point 3:***

In your ethics statement in the Methods section and in the online submission form, please provide additional information about the data used in your retrospective study. Specifically, please ensure that you have discussed whether all data were fully anonymized before you accessed them and/or whether the IRB or ethics committee waived the requirement for informed consent. If patients provided informed written consent to have data from their medical records used in research, please include this information.

***Response*:** This study was approved by the Northwestern University IRB and granted a waiver of consent. Data was provided by the Northwestern Medicine Electronic Data Warehouse and included protected health information. We have updated the ethics statement in the Methods section and the online submission form accordingly.

***Editor, Point 4:***

In your Methods section, please provide additional information about the participant selection method and the demographic details of your participants. Please ensure you have provided sufficient details to replicate the analyses such as:
a) the recruitment date range (month and year),
b) the institutions where patients were from,
c) a statement as to whether your sample can be considered representative of a larger population

***Response*:** We appreciate the opportunity to clarify. We have revised our methods section to include the requested details and are happy to make any additional changes if requested by the Editor.

***Editor, Point 5:***

We note that you have indicated that data from this study are available upon request. PLOS only allows data to be available upon request if there are legal or ethical restrictions on sharing data publicly. For information on unacceptable data access restrictions, please see <http://journals.plos.org/plosone/s/data-availability#loc-unacceptable-data-access-restrictions>.

***Response***: Thank you for the opportunity to clarify. In accordance with PLOS requirements, we are unable to share a de-identified dataset due to the potential of identifying sensitive patient information. We have clarified this in our cover letter.

***Editor, Point 6:***

In your revised cover letter, please address the following prompts:

 a) If there are ethical or legal restrictions on sharing a de-identified data set, please explain them in detail (e.g., data contain potentially identifying or sensitive patient information) and who has imposed them (e.g., an ethics committee). Please also provide contact information for a data access committee, ethics committee, or other institutional body to which data requests may be sent.

b) If there are no restrictions, please upload the minimal anonymized data set necessary to replicate your study findings as either Supporting Information files or to a stable, public repository and provide us with the relevant URLs, DOIs, or accession numbers. Please see <http://www.bmj.com/content/340/bmj.c181.long> for guidelines on how to de-identify and prepare clinical data for publication. For a list of acceptable repositories, please see <http://journals.plos.org/plosone/s/data-availability#loc-recommended-repositories>.

We will update your Data Availability statement on your behalf to reflect the information you provide.

***Response:*** Thank you for this information. We have updated our cover letter accordingly.

***Editor, Point 7:***

PLOS requires an ORCID iD for the corresponding author in Editorial Manager on papers submitted after December 6th, 2016. Please ensure that you have an ORCID iD and that it is validated in Editorial Manager. To do this, go to ‘Update my Information’ (in the upper left-hand corner of the main menu), and click on the Fetch/Validate link next to the ORCID field. This will take you to the ORCID site and allow you to create a new iD or authenticate a pre-existing iD in Editorial Manager. Please see the following video for instructions on linking an ORCID iD to your Editorial Manager account: <https://www.youtube.com/watch?v=_xcclfuvtxQ>

***Response***: We have added the ORCID iD for Dr. Badreldin in the submission portal (0000-0002-6341-3350).

**REVIEWER 1**

***Reviewer 1, Point 1:***

Since authors have studied only vaccination data of 11/2011-11/2012 (“pre-guideline”) and 12/2012-12/2015 (“post guideline”) that is form single care center only, therefore authors should include more data to their study.

***Response:*** Thank you for this feedback. We believe that our sample size is large enough to detect meaningful differences across groups. However, we agree that future research is warranted in broader settings and with larger sample sizes. We have included this in our limitations section.

***Reviewer 1, Point 2:***

Figure’s quality is not good for publication. Authors may improve the quality.

***Response***: We apologize for this inconvenience. We have updated file type and the quality of our figures in accordance with PLOS ONE requirements.

***Reviewer 1, Point 3:***

In figure 1, authors should check the total number of individuals included in their study as sum of pre-guidelines and post-guidelines individuals (684+1610= 2294) is more than total number of individuals (2288).

**Response**: Thank you for bringing this to our attention. We have carefully reviewed and updated the values in Figure 1.

***Reviewer 1, Point 4:***

Why 2012 ACIP guidelines could not address racial disparities in vaccine uptake, authors may suggest their view to improve these guidelines further.

***Response:*** We appreciate this suggestion and agree that efforts to alleviate racial disparities in prenatal vaccine uptake are of utmost importance. We have revised our manuscript to include a discussion on the importance of acknowledging and addressing racial disparities in vaccine uptake.
